# A systematic review of Clinical Practice Guidelines for the development of the WHO's Package of Interventions for Rehabilitation: focus on schizophrenia

**DOI:** 10.3389/fpubh.2023.1215617

**Published:** 2023-08-15

**Authors:** Riccardo Serra, Yasaman Etemadi, Marieke van Regteren Altena, Corrado Barbui, Lorenzo Tarsitani

**Affiliations:** ^1^Department of Human Neurosciences, Sapienza University of Rome, Rome, Italy; ^2^Section of Psychiatry, Department of Neuroscience, Biomedicine and Movement Sciences, WHO Collaborating Centre for Research and Training in Mental Health and Service Evaluation, University of Verona, Verona, Italy; ^3^Department of Neurosciences, Center for Public Health Psychiatry, KU Leuven University, Leuven, Belgium; ^4^Sensory Functions, Disability and Rehabilitation Unit, Department of Noncommunicable Diseases, World Health Organization, Geneva, Switzerland; ^5^Department of Mental Health and Substance Use, World Health Organization, Geneva, Switzerland

**Keywords:** schizophrenia, psychosis, rehabilitation, systematic review, World Health Organization (WHO)

## Abstract

**Background:**

The identification of interventions for rehabilitation and related evidence is a crucial step in the development of World Health Organization's (WHO) Package of Interventions for Rehabilitation (PIR). Interventions for rehabilitation may be particularly relevant in schizophrenia, as this condition is associated with a high risk of disability, poor functioning, and lack of autonomy. Aiming to collect evidence for the WHO PIR, we conducted a systematic review of Clinical Practice Guidelines (CPG) on interventions for rehabilitation of schizophrenia.

**Methods:**

Methods for the systematic identification and critical appraisal of CPG were developed by WHO Rehabilitation Programme and Cochrane Rehabilitation under the guidance of WHO's guideline review committee secretariat. The Appraisal of Guidelines for Research & Evaluation Instrument (AGREE II) was used to evaluate the methodological quality of identified CPG.

**Results:**

After full text screening, nine CPG were identified, for a total of 130 recommendations. Three were excluded because their total AGREE-II scores were below cut-off. Six CPG were approved by the Technical Working Group and included for data extraction. Only one CPG with specific focus on rehabilitation of schizophrenia was retrieved. Other CPG were general, including some recommendations on rehabilitation. Some CPG gave no indications on the assessment of rehabilitation needs. Discrepancies were detectable, with different CPG emphasizing different domains. Most recommendations addressed “symptoms of schizophrenia,” while “community and social life” was targeted by few recommendations. International CPG were often conceptualized for high-income countries, and CPG accounting for their implementation in lower income contexts were scarce. Quality of evidence was high/moderate for 41.54% (*n* = 54) of the recommendations, and very low only in two cases (1.52%). *N* = 45 (34.62%) were based on experts' opinion.

**Conclusions:**

The concepts of recovery and rehabilitation in schizophrenia are relatively new in medical sciences and somewhat ill-defined. An unbalanced distribution in the domains addressed by available CPG is therefore understandable. However, the need for more focus in some areas of rehabilitation is obvious. More clarity is also required regarding which interventions should be prioritized and which are more feasible for global implementation in the rehabilitation of schizophrenia.

## 1. Introduction

The World Health Organization (WHO) has the strategic priority of achieving Universal Health Coverage (UHC), that means “all people receive quality health services that meet their needs without being exposed to financial hardship in paying for the services” (1). UHC includes rehabilitation interventions among the services to be provided. As part of the WHO Rehabilitation 2030 call for action (2), the WHO Rehabilitation Programme has been developing a Package of Interventions for Rehabilitation (PIR) to support ministries of health in integrating rehabilitation services into health systems (3).

The development of the PIR takes a stepwise approach composed of five consecutive steps: (a) selection of health conditions considering amenability to and the need for rehabilitation, (b) identification of evidence-based interventions for rehabilitation, (c) development of the package with agreement on interventions along with resource requirement descriptions, (d) external peer review, and (e) production of the alpha version of the PIR (3). This paper reports on the second step, which includes the identification of interventions for rehabilitation and related evidence for the health conditions selected in the first step. Under WHO's Guideline Review Committee Secretariat guidance, the WHO Rehabilitation Programme and Cochrane Rehabilitation developed the corresponding methods. Underpinning evidence for the development of the PIR includes a series of systematic reviews of Clinical Practice Guidelines (CPG) for different health conditions selected. Interventions and related evidence identified from these CPG undergo a consensus process to select essential interventions in step 3, to be included in the final PIR. Information related to the provision of the interventions will be added. All information undergoes a review process before developing the final version of the PIR.

Schizophrenia has been selected as one of the priority targets of the PIR. Although not as common as other disabling conditions possibly affecting humans, psychosis resembles the historical antonomasia of mental disability due to its profound long-term effects on individuals, families, and societies. One in 135 people (0.72% of the population), or over 56 million people worldwide, are affected by schizophrenia (4). Proportions are larger in the adult population, due to an incidence peak between the age of 15 and 25 years (4–6). Schizophrenia affects the general population equally, with no substantial difference between males and females, or among urban, rural, and mixed sites (4). Schizophrenia may have a highly detrimental effect on the life of a person and their family, impacting the individual's existence on multiple levels. Especially if left untreated, this condition may progressively deteriorate the personal, familial, relational, educational, and occupational possibilities of a person, accounting for 13.4 million years of life lived with disability globally (6). Profound stigmatization for their condition and their symptoms is the rule rather than the exception for people with schizophrenia across countries, with consequences in terms of social exclusion, isolation, and frequent human rights violation (7–9). This deeply disabling condition is also related to higher odds of dying prematurely due to preventable causes, with a life expectancy of 20 years less than the general population (10, 11).

Despite the high risk of severe, lifelong problems that affect every area of life, around 15% of people with schizophrenia will eventually reach nearly complete recovery (12). The concept of recovery and the concept of rehabilitation share blurred boundaries, and need to be seen as two faces of the same coin. As Anthony (13) well-depicted with regards to recovery from mental illness “Recovery is what people with disabilities do. Treatment, case management, and rehabilitation are what helpers do to facilitate recovery.” Recovery may be related to several interventions, and an early approach is considered pivotal, as highlighted in the recent WHO World Mental Health Report (WMHR), which also emphasized that an holistic multidisciplinary rehabilitation process is key in this population, especially in the long term (14–16). According to WHO, “*Rehabilitation helps a child, adult or older person to be as independent as possible in everyday activities and enables participation in education, work, recreation and meaningful life roles such as taking care of family*” (17).

Finding a single definition of rehabilitation is challenging, and the differences between rehabilitation for physical and mental health problems add to the difficulty. Since rehabilitation services and techniques have started developing, they sprouted in a multitude of different approaches and theories, sometimes drifting from one another, and leaving a doubt on what rehabilitation actually is. A comprehensive perspective on what constitutes rehabilitation for mental disorders was provided in the consensus statement on psychosocial rehabilitation by the WHO in collaboration with the World Association for Psychosocial Rehabilitation in 1996 (18). In this document rehabilitation is described as “a process that facilitates the opportunity for individuals—who are impaired, disabled or handicapped by a mental disorder—to reach their optimal level of independent functioning in the community,” which is inextricably founded on the individual, the service, and the environment. These concepts kept evolving, broadening the focus not only on “functioning” as part of the social apparatus but also on regaining roles and meaning in each individual life. For example, the Person-Centered Psychiatric Rehabilitation model by Farkas and colleagues (19) states: “*Without a process committed to supporting chosen roles and settings, functioning may be improved, but the individual's vision of a meaningful life may still not be achieved. Rehabilitation, therefore, works with social relationships, work, leisure, family life, higher education, and other student pursuits, using interventions that focus on increasing competencies or skills and providing environmental supports*.” Similarly, regarding the rehabilitation of mental illness, the National Institute for Health and Care Excellence states: “*the guiding principle is the belief that it is possible for someone to regain a meaningful life, despite serious mental illness… it refers to someone achieving the best quality of life they can, while living and coping with their symptoms. It is an ongoing process whereby the person is supported to build up their confidence and skills and resilience, through setting and achieving goals to minimize the impact of mental health problems on their everyday life*.” Specifically for the WHO-PIR development, the definition of rehabilitation, used for both physical and mental health conditions, was: “Interventions aiming at improving functioning across all levels of functions (including body functions, activity, and participation) without targeting the cause of the health condition.” Regardless of the specific definition, health systems around the world are urged to put into place appropriate rehabilitation services for people with mental health conditions (16), taking into account the specific individuals, their background and social context, and the available individual and community resources.

Many interventions have shown to be effective as rehabilitation strategies for schizophrenia in well-designed randomized controlled trials. For example, a systematic review on rehabilitation interventions to promote recovery from schizophrenia, identified a total of 80 studies (nine of which were meta-analyses) which consistently provide evidence of the efficacy of interventions such as Cognitive Remediation, Family Psychoeducation, Social Skills Training, and Cognitive Therapy (20). Furthermore, although the healthcare gaps among countries around the world (21, 22), some of these interventions have been successfully implemented in low- and middle-income countries (23, 24). However, given the wide heterogeneity of existing interventions that can be considered as interventions for rehabilitation, policy makers, stakeholders, professionals, and service users may find it difficult to navigate through alternative intervention options and make informed decisions. Some CPG have been developed to organize and summarize existing evidence, but the field of rehabilitation in schizophrenia is still fragmented and somewhat contradictory in terms of recommendations. Therefore, as a base for the WHO PIR, we conducted a systematic review of existing CPG relevant to rehabilitation of people with schizophrenia.

## 2. Methods

**Figure 1:**
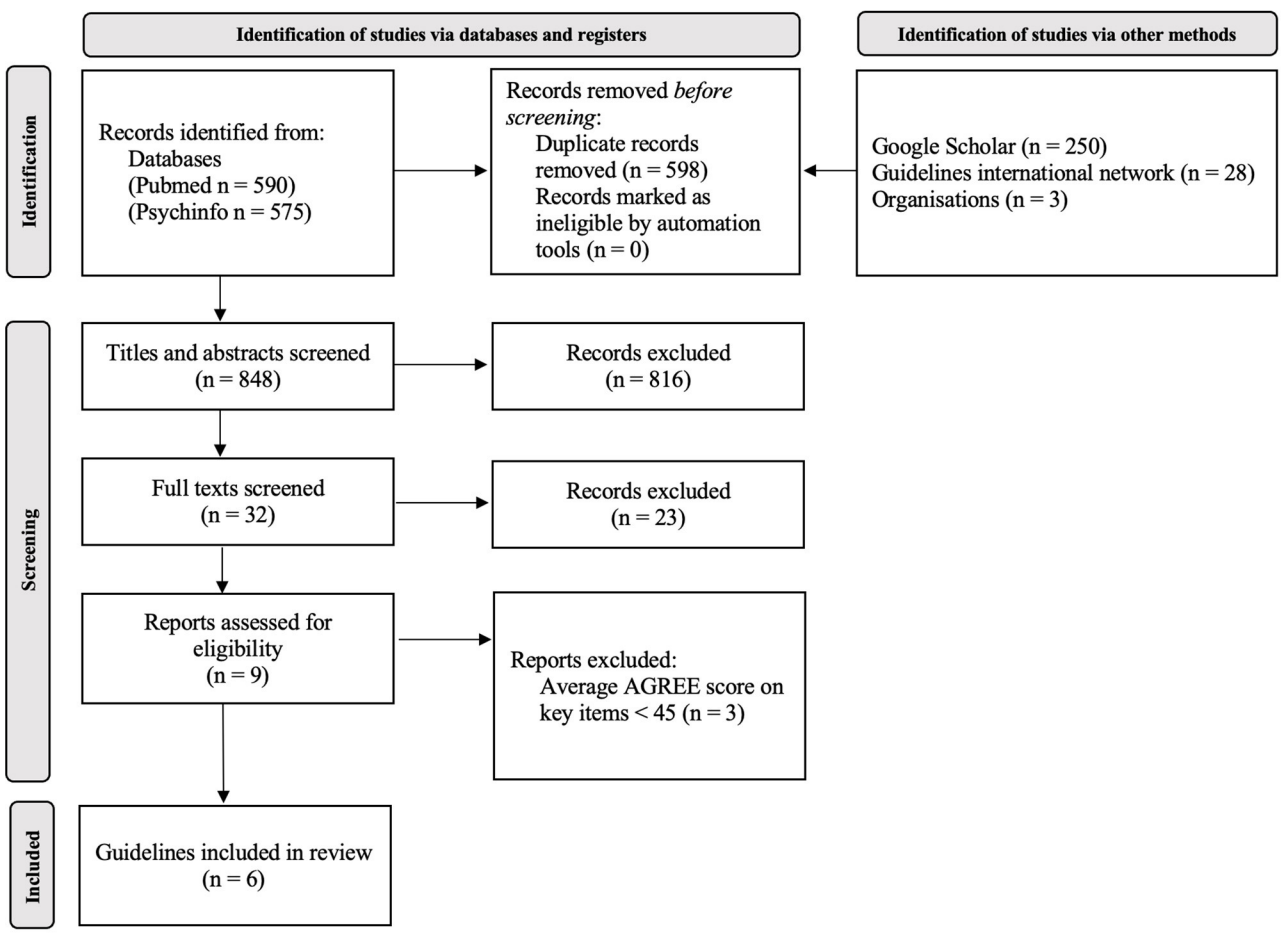
Results of the search and screening process for identification of suitable guidelines.

This systematic review of CPG has been developed in full compliance with the methodology presented in the introductory WHO PIR protocol of research (3). These stages have been followed (Figure 1):

- Systematic literature search: CPG have been searched in the following databases: Guidelines International Network (GIN), Scotland's Guidelines International Network (G-I-N), United Kingdom's National Institute for Health and Care Excellence (NICE), Australia's National Health and Medical Research Council (NHMRC), Scottish Intercollegiate Guidelines Network (SIGN), Canadian Medical Association Infobase of Clinical Practice Guidelines (CPG Infobase), New Zealand Guidelines Group (NZGG), United States' National Guideline Clearinghouse (NGC), United Kingdom's eGuidelines, Psychinfo, Pubmed, Google scholar (first 250 results only). The search string used was: “(Guideline^*^) AND (schizophrenia OR Psychosis) AND (psychosocial OR rehabilitation OR recovery OR functioning).” A filter to include only publications in English language was applied. First search was performed in April 2020, and then it was re-run in March 2021. Only publications from 2010 were considered, aiming to include up-to-date recommendations only.
- Independent title and abstract screening of the retrieved manuscripts by two authors. The screening aimed at retrieving only CPG on schizophrenia. For the title and the abstract screening, inclusion criteria were as follows: “Does the manuscript present a guideline?” “Is the guideline specifically developed for the health condition of interest?” “Is the guideline a guideline on rehabilitation interventions?” “Is the guideline not older than 10 years?”. Only if all four questions were answered with “Yes” the screening author would endorse “Yes” for that reference. If at least one of the two authors had endorsed “Yes” that reference would proceed to the next step. The same was applied for both title and abstract screening.
- Independent full text screening. The full text screening applied the same previous criteria plus two more as follows: “Is it clear that there is no conflict of interest?” “Is information on the strength of the recommendation provided?”. If all questions were answered “yes” then the author would endorse “yes” for that reference. If at least one of the two authors had endorsed “Yes” that reference would be included in the next step.
- Independent evaluation of the CPG quality with the “Appraisal of Guidelines for Research and Evaluation” (AGREE II) tool by two authors (RS and YE) (25): a specific focus has been given to items 7, 8, 12, and 22 where the average result had to be >2 (AGREE/4), and to the items 4, 7, 8, 10, 12, 13, 15, 22, and 23 whose average sum score had to be >45 (AGREE/9). Evaluation discrepancies were confronted and an average grade of the two separate evaluation was applied.
- CPG fitting AGREE II criteria were given a final consensus for inclusion according to the using the WHO prioritization criteria (quality, publication time, multiprofessionality, comprehensiveness) (3).
- Data extraction was independently performed by two authors using a standardized form which includes information on the type of recommendation, dosage, target group, strength of recommendation and quality of the background evidence in support to the recommendation.

Topics/areas considered by the selected CPG into each main type of recommendation (assessment and intervention) have been extracted. Recommendations regarding the management of the acute phase of illness, recommendations on medications and side-effects, as well as service recommendations were excluded, therefore leading to a selection of non-drug-related recommendations on the long-term course of the treatment. The topics (functioning domains) from different CPG have been compared independently by two authors and integrated. If needed, agreement by discussion was reached involving a third author. The process has been repeated for all the CPG until final agreement on the topics was reached.

## 3. Results

Results of the literature search are shown in Figure 1, in agreement with the PRISMA statement (26). A total of nine CPG were selected on the basis of abstract and full-text screening. Details on the abstract and full-text screening are shown in Supplementary Table 1. As shown in Table 1, three of these were excluded due to a total AGREE-II score <45 (27–29), leading to the inclusion of six guidelines (25). The six CPG were approved by the Technical Working Group and included for data extraction:

– *German Association for Psychiatry, Psychotherapy and Psychosomatics Revised S3 guidelines on schizophrenia* (DGPPN) (30).
– *The American Psychiatric Association Practice Guideline for the Treatment of Patients with Schizophrenia* (APA) (31).
– *Scottish Intercollegiate Guidelines Network*: *management of schizophrenia* (SIGN) (32).
– *National Institute for Health and Care Excellence Psychosis and schizophrenia in children and young people: recognition and management* (NICE-1) (33).
– *National Institute for Health and Care Excellence Psychosis and schizophrenia in adults: prevention and management* (NICE-2) (34).
– *National Institute for Health and Care Excellence Rehabilitation for adults with complex psychosis* (NICE-3) (35).

**Table 1:** Main characteristics of included and excluded guidelines of rehabilitation interventions for people with schizophrenia and related psychosis.

**Guideline**	**AGREE ratings**					**Included (yes/no)**	**Subject**	**Publication date**
	***Average of key items***				***Average of summary value of items 4, 7, 8, 10, 12, 13, 15, 22, 23***			
	***7***	***8***	***12***	***22***				
**Included**								
1) German Association for Psychiatry, Psychotherapy and Psychosomatics: S3 Guideline for Schizophrenia	6.5	6.5	7	7	58.5	Yes	Diagnosis, treatment, rehabilitation, and care of patients with schizophrenia	2019
2) American Psychiatric Association: Practice guideline for the treatment of patients with schizophrenia	6.5	5.5	6.5	6.5	53	Yes	Treatment of patients with schizophrenia	2020
3) National Institute of Health and Care Excellence (NICE): Psychosis and schizophrenia in adults: prevention and management. CG178	6	6	5	4.5	52	Yes	Treatment and management of psychosis and schizophrenia	2016
4) National Institute of Health and Care Excellence (NICE): Psychosis and schizophrenia in children and young people: recognition and management. CG155	6	6	5	4.5	52	Yes	Recognition and management of psychosis and schizophrenia in children and young people	2016
5) National Institute for Health and Care Excellence (NICE): Rehabilitation for adults with complex psychosis. NICE Guideline 181	5	5	4	4	45	Yes	Rehabilitation for adults with complex psychosis	2020
6) Scottish Intercollegiate Guidelines Network (SIGN): Management of schizophrenia. SIGN 131	5.5	4.5	7	4.5	49.5	Yes	Care and treatment of adults with schizophrenia	2013
**Excluded**								
Royal Australian and New Zealand College of Psychiatrists: Clinical practice guidelines for the management of schizophrenia and related disorders	4	3	6	3	40	No	Care of people with schizophrenia and related disorders	2016
Stubbs B, Vancampfort D, Hallgren M et al. European Psychiatric Association (EPA): EPA guidance on physical activity as a treatment for severe mental illness: a meta-review of the evidence and Position Statement from the European Psychiatric Association (EPA), supported by the International Organization of Physical Therapists in Mental Health (IOPTMH)	6	6	4	6	41.5	No	Physical activity in the treatment of schizophrenia-spectrum disorders, major depressive disorders and bipolar disorder	2018
European Psychiatric Association (EPA): guidance on the early intervention in clinical high-risk states of psychoses	6	6	3.5	4	40.5	No	Evidence-based recommendations on early intervention in clinical high-risk states of psychosis	2015

In addition to the selected CPG, relevant WHO guidelines including mhGAP Evidence-based recommendations for management of psychosis and bipolar disorders in non-specialized health settings as well as management of physical health conditions in adults with severe mental disorders were included for the development of PIR. However, these resources have not been included in this review.

The topics (functioning domains) extracted from included CPG are presented by type of recommendation (assessment/intervention) in Table 2. The original recommendations from included CPG, related quality of evidence (QoE) and the strength of recommendation (SoR) are presented in Table 3. As seen in Table 2, different topics of intervention are resumed in the first column, and each line presents the number of interventions on that specific topic for each included CPG. Recommendations are reported multiple times if the original recommendation included multiple target outcomes or multiple interventions for the same target. SoR and QoE are presented in a standardized fashion to avoid discrepancies in terms used among CPG. Rationale applied to this classification and original classifications are presented in Supplementary Table 2. Among all CPG, only NICE-3 had a specific focus on the rehabilitation of schizophrenia (35).

**Table 2:** Number of recommendations per topic (functioning domain) and recommendation type.

**Topics/functioning domain**	**Selected guidelines**						**Selected guidelines**						**Total**
	**DGPPN**	**APA**	**NICE1**	**NICE2**	**NICE3**	**SIGN**	**DGPPN**	**APA**	**NICE1**	**NICE2**	**NICE3**	**SIGN**	
	**Assessment recommendations**						**Intervention recommendations**						
a) Activities of daily living	–	–	1	–	1	–	–	–	–	–	4	–	6
b) Self-management	–	–	–	–	–	–	3	2	2	–	2	–	9
c) Career and family support	1	–	1	1	–	–	1	–	2	–	1	–	7
d) Interpersonal interactions and relations	–	–	–	–	1	–	1	1	–	1	2	1	7
e) Community and social life	–	–	–	–	–	–	1	–	–	–	3	–	4
f) Education and vocation	–	–	1	3	1	–	3	1	2	2	4	–	17
g) Exercise and fitness	–	–	–	–	1	–	1	–	–	1	1	1	5
h) Lifestyle modification	1	–	1	–	1	–	1	–	1	1	3	2	11
i) Speech, language and communication	–	–	–	–	1	–	–	–	–	1	–	–	2
j) Sexual functions and intimate relations	1	–	–	–	2	–	–	–	–	–	1	1	5
k) Mental/emotional functions	2	–	–	2	1	–	2	–	–	–	–	1	8
l) Mental/cognitive functions	1	–	–	–	1	–	2	1	–	1	2	1	9
m) Symptoms of psychosis	–	–	–	–	1	–	11	3	8	9	5	3	40
Total	**6**	**0**	**4**	**6**	**11**	**0**	**26**	**8**	**15**	**16**	**28**	**10**	**130**

DGPPN, German Association for Psychiatry, Psychotherapy and Psychosomatics Revised S3 guidelines on schizophrenia; APA, The American Psychiatric Association Practice Guideline for the Treatment of Patients With Schizophrenia; SIGN, Scottish Intercollegiate Guidelines Network: management of schizophrenia; NICE-1, National Institute for Health and Care Excellence Psychosis and schizophrenia in children and young people: recognition and management; NICE-2, National Institute for Health and Care Excellence Psychosis and schizophrenia in adults: prevention and management; NICE-3, National Institute for Health and Care Excellence Rehabilitation for adults with complex psychosis.

**Table 3:** Recommendations from selected CPG with reported strength of recommendation and quality of the evidence.

**Original recommendation**	**Ref**	**SoR**	**QoE**
**a) Activities of daily living**			
**Assessment of activities of daily living**			
Routinely **record the daytime activities** of people with psychosis or schizophrenia in their care plans, including occupational outcomes	3	S	H
Offer people a comprehensive biopsychosocial needs assessment by a multidisciplinary team within 4 weeks of entering the rehabilitation service. Include the following as part of the **comprehensive assessment:** · current **skills in activities of daily living**	5	S	U.K.
**ADL training**			
Staff should build on people's strengths and encourage hope and optimism by: ·**help**ing people to **gain skills** to **manage** both their **everyday activities** and their mental health, including moving toward self-management of medication (see the recommendations on helping people to manage their own medicines)	5	S	L
**Provide activities** to help people with complex psychosis develop and **maintain daily living skills** such as self-care, laundry, shopping, budgeting, using public transport, cooking and communicating (including using digital technology)	5	S	M
**Support** people to **engage in activities** to **develop** or **improve their daily living skills by**: · making a plan with each person that focuses on their needs and regularly reviews their goals ·**providing activities** they **enjoy** and that motivate them · enabling them to practice their skills in risk-managed real life, such as kitchens and laundry rooms, wherever possible	5	S	M
Offer people the chance to be involved in a range of **activities that they enjoy**, tailored to their level of ability and wellness	5	S	M
**b) Self management**			
**Peer support**			
Consider **peer support** for people with psychosis or schizophrenia to help **improve service user experience** and quality of life. Peer support should be delivered by a trained peer support worker who has recovered from psychosis or schizophrenia and remains stable. Peer support workers should receive support from their whole team, and support and mentorship from experienced peer workers	3	C	L
# **Peer-to-peer concepts** may be considered for people with schizophrenia with the aim to achieve **greater optimism and recovery Psychoeducational approaches** based on the peer-to-peer model may be considered for patients, family members and close confidants to enable alternative paths, have a positive effect on the growth of knowledge and concept of the illness and reduce subjective distress	1		E.O.
Staff should build on people's strengths and encourage hope and optimism by: · providing opportunities for **sharing experiences with peers**	5	S	L
**Psychoeducation**			
APA recommends that patients with schizophrenia receive **psychoeducation**	2	S	M
To improve treatment outcome and illness course, as part of an overall treatment plan we recommend offering **structured psychoeducation** for a sufficient time and, if possible, in **groups** to people with schizophrenia. We recommend involving family members and close confidants in the psychoeducational intervention	1	S	H
**Other interventions**			
APA suggests that patients with schizophrenia receive interventions aimed at developing **self-management skills** and enhancing person- oriented recovery	2	C	L
Consider a **manualized self-management programme** delivered face- to-face with service users, as part of the treatment and management of psychosis or schizophrenia	3	C	H
Staff should build on people's strengths and encourage hope and optimism by: ·**help**ing people to **gain skills** to manage both their everyday activities and their mental health, including moving toward **self-management** of medication	5	S	L
# We suggest that all support approaches have the goal to promote the service user's social connections. We suggest that **self-help** by the service user, as well as family members and close confidants, is **encouraged**, service users' **self- confidence** is **strengthened** and their wish for **information and involvement** in treatment decisions is strongly **supported**	1		E.O.
**c) Carer and family support**			
**Assessment of carer and family needs**			
# Family members and other close confidants of people with schizophrenia are exposed to significant emotional stress. At the same time, family members and close confidants are the most important sources of social support for service users in the long term. Therefore, family members and close confidants should be viewed as also being affected by the illness. We recommend offering them information about schizophrenia, while maintaining confidentiality. We recommend regularly **asking** family members and close confidants about **their need for support**. Depending on individual needs, we recommend offering individual support in dealing with the emotional stress	1		E.O.
# Offer **carers** of people with psychosis or schizophrenia **an assessment** (provided by mental health services) of their own **needs** and discuss with them their strengths and views. Develop a care plan to address any identified needs, give a copy to the carer and their GP and ensure it is reviewed annually	3	S	E.O.
# Advise parents and carers about their right to a **formal carer's assessment of their own physical and mental health needs**, and explain how to access this	4	S	E.O.
**Caregiver training**			
# **Advise carers** about their statutory right to a formal carer's assessment provided by social care services and explain how to access this	3	S	E.O.
Offer a **carer-focused education and support programme**, which may be part of a family intervention for psychosis and schizophrenia, as early as possible to all carers. The intervention should: · be available as needed · have a positive message about recovery	3	S	H
# Family members and other close confidants of people with schizophrenia are exposed to significant emotional stress. At the same time, family members and close confidants are the most important sources of social support for service users in the long term. Therefore, family members and close confidants should be viewed as also being affected by the illness. We recommend **offering** them **information** about schizophrenia, while maintaining confidentiality. We recommend regularly asking family members and close confidants about their need for sup- port. Depending on individual needs, we recommend **offering** individual **support** in dealing with the emotional stress	1		E.O.
**Give families and carers information** about **support services** in their area that can **address their emotional, practical and other needs** (this is particularly important if the person is accessing rehabilitation services for the first time)	5	S	M
**d) Interpersonal interactions and relationships**			
**Assessment of interpersonal interactions and relationships**			
Offer people a comprehensive biopsychosocial needs assessment by a multidisciplinary team within 4 weeks of entering the rehabilitation service. Include the following as part of the **comprehensive assessment:** ·**current social network**, including any caring responsibilities	5	S	U.K.
**Structured group activities**			
Offer **structured group activities (**social, leisure or occupational) aimed at improving **interpersonal skills**. These could be peer-led or peer-supported and should be offered: · daily in inpatient rehabilitation services · at least weekly in community settings	5	S	M
**Psychosocial support**			
# **Enable** the person to **maintain links with** their **home community** by: ·**support**ing them to **maintain relationships with family and friends**, for example, by finding ways to help with transport · helping them to stay in touch with social and recreational contacts · helping them to keep links with employment, education and their local community	5	S	E.O.
**Social skills training**			
**Social skills training** may be considered for individuals diagnosed with schizophrenia who have persisting **problems related to social skills**	6	C	H
APA suggests that patients with schizophrenia who have a therapeutic goal of enhanced **social functioning** receive **social skills training**	2	C	L
We recommend offering **social skills training** if there are relevant **deficits in social skills** and in case of persistent negative symptoms. Social skills training should last for several months and be supplemented with tasks for transferring skills to everyday life	1	S	H
**Art therapy**			
# If **arts therapies** are considered, they should be provided by Health Professions Council (HPC) registered arts therapists, with experience of working with children and young people with psychosis or Schizophrenia. The intervention should be provided in groups unless difficulties with acceptability and access and engagement indicate otherwise. Arts therapies should combine psychotherapeutic techniques with activity aimed at promoting creative expression, which is often unstructured and led by the child or young person. **Aims of arts therapies** should include: ·**enabling** children and young people with psychosis or schizophrenia to experience themselves differently and **to develop new ways of relating to others**	4	C	E.O.
**e) Participation in community and social life**			
**Participation focused interventions**			
**Programmes** to **engage people** in **community activities** should: · be flexible and make reasonable adjustments to accommodate the person's illness and fluctuating needs · be individualized · develop structure and purpose in the person's day ·**aim to increase** their sense of identity, belonging and **social inclusion in the community** · involve peer support · recognize people's skills and strengths	5	S	H
Staff should build on people's strengths and encourage hope and optimism by: ·**help**ing them **find meaningful occupations** (including work, leisure or education) and build support networks using voluntary, health, social care and mainstream resources ·**help**ing people to **gain skills** to manage both their **everyday activities** and their mental health, including moving toward self-management of medication	5	S	H
# **Enable** the person to **maintain links** with their **home community** by: · supporting them to maintain relationships with family and friends, for example, by finding ways to help with transport •**help**ing them to **stay in touch** with **social and recreational contacts** · helping them to keep links with employment, education and their local community	5	S	E.O.
# We suggest that **all support approaches have the goal to promote** the service user's **social connections**. We suggest that self-help by the service user, as well as family members and close confidants, is encouraged, service users' self- confidence is strengthened and their wish for information and involvement in treatment decisions is strongly supported	1		E.O.
**f) Education and vocation**			
**Educational and vocational assessment**			
# Routinely **record** the **daytime activities** of children and young people with psychosis or schizophrenia in their care plans, including **educational** and occupational **outcomes**	4	S	E.O.
# Routinely **record the daytime activities** of people with psychosis or schizophrenia in their care plans, including **occupational outcomes**	3	S	E.O.
# Routinely **record** the **daytime activities** of children and young people with psychosis or schizophrenia in their care plans, including educational and **occupational outcomes**	4	S	E.O.
Offer people a comprehensive biopsychosocial needs assessment by a multidisciplinary team within 4 weeks of entering the rehabilitation service. Include the following as part of the **comprehensive assessment:** ·**occupational and educational history**, including educational attainment and reason for leaving any employment	5	S	U.K.
# **Liaise** with the child or young person's school and with their parents or carers, subject to consent, **to determine** whether a **special educational needs** assessment is necessary. If it is agreed that this is needed, explain to parents or carers how to apply for an assessment and offer support throughout the process	4	S	E.O.
**Vocational counseling, training, or support**			
Consider providing a cognitive remediation intervention alongside **vocational rehabilitation** services	5	C	H
For people who are not ready to return to paid employment, consider alternatives such as **transitional employment schemes** and volunteering	5	C	H
# Offer supported employment programmes to people with psychosis or schizophrenia who wish to find or return to work. Consider other occupational or educational activities, including **pre-vocational training**, for people who are unable to work or unsuccessful in finding employment	3	S	E.O.
We suggest making options also available to people with schizophrenia that take the approach **“train first**—then place.” Such options are especially important in the subgroup of patients who do not want to be immediately employed in the general labor market. The aim is supported **employment** in the general labor market	1	S	M
Offer people a range of **educational and skill development opportunities**, for example, recovery colleges and mainstream adult education settings, which **build confidence** and may lead **to qualifications** if the person wishes	5	S	H
# Help the child or young person to continue their **education**. **Contact** the school or college, subject to consent, to **ask for additional educational support** if their performance has been affected	4	S	E.O.
**Supported employment programme**			
As part of **occupational rehabilitation**, we recommend offering **programmes** to people with schizophrenia who want to work in the general labor market that aim to quickly place them directly in a job in the general labor market and provide the necessary **support (supported employment)**	1	S	H
# Offer **supported employment programmes** to people with psychosis or schizophrenia who wish to find or return to work. Consider other occupational or educational activities, including pre-vocational training, for people who are unable to work or unsuccessful in finding employment	3	S	E.O.
Provide **supported employment programmes** for those young people with psychosis or schizophrenia above compulsory school age who wish to return to work or find employment. Consider other work-related activities and programmes when individuals are unable to work or are unsuccessful in their attempts to find employment	4	S	L
APA recommends that patients with schizophrenia receive **supported employment services**	2	S	M
For people who would like to work toward mainstream employment, consider referring them to **supported employment** that uses the Individual Placement and Support approach	5	C	H
The efficacy of approaches that follow the principles of **supported employment** can be increased by cognitive remediation. We suggest that such remediation is therefore performed, depending on the individual need	1	C	M
**g) Excercise and fitness**			
**h) Lifestyle modification**			
**Assessment of lifestyle risk factors**			
The initial physical health check in the **comprehensive assessment** by the rehabilitation service should include: · body mass index · waist circumference · pulse and blood pressure · smoking, alcohol and illicit substance use · nutritional status, **diet, and level of physical activity**	5	S	U.K.
Offer people in rehabilitation services a **routine physical health** check at least annually. The annual physical health check should include: · body mass index · waist circumference · pulse and blood pressure · smoking, alcohol and illicit substance use · nutritional status, **diet and level of physical activity**	5	S	L
**Routinely monitor weight**, and cardiovascular and metabolic indicators of morbidity in people with psychosis and schizophrenia. These should be audited in the annual team report	3	S	H
# We recommend that in addition to the principles applied in adult patients, **side-effect monitoring in children and adolescents** (<18 years old) receiving antipsychotics takes into account the specific characteristics of this age group. These include: · Sex- and age-dependent assessment of side effects (in particular motor side effects) · Consideration of the high sensitivity for motor side effects · Consideration of the differences in the objective and subjective perception of side effects · Effect of elevated prolactin levels on sexual development · Effect of treatment on height and **weight development**, with regular follow-up monitoring of these two important physical parameters · Compared with adults, more frequent monitoring of possible metabolic side effects	1		E.O.
**Education and advice on healthy lifestyle**			
**Offer** people with psychosis or schizophrenia who smoke **help to stop smoking**, even if previous attempts have been unsuccessful. Be aware of the potential significant impact of reducing cigarette smoking on the metabolism of other drugs, particularly clozapine and olanzapine	3	S	M
# **Promote** good physical health, including **healthy eating**, exercise **and smoking cessation**	4	S	E.O.
# **Promote** good physical health, including healthy eating, **exercise** and smoking cessation	4	S	E.O.
Offer people who smoke **help to stop smoking**, even if previous attempts have been unsuccessful	5	S	L
People with psychosis or schizophrenia, especially those taking antipsychotics, should be offered a combined healthy eating and **physical activity** programme by their mental healthcare provider	1	S	H
People with psychosis or schizophrenia, especially those taking antipsychotics, should be offered a combined **healthy eating** and physical activity programme by their mental healthcare provider	1	S	H
Offer people a combined healthy eating and **physical activity programme and support** them to take part in it	5	S	L
Offer people a combined **healthy eating** and physical activity programme **and support** them to take part in it	5	S	L
**Give** people clear and accessible **information** about any **health risks** related to their: · medicines (side effects) · lifestyle, including: · diet and physical activity · smoking, alcohol or illicit substance use—oral hygiene · bone health · sexual and reproductive health	5	S	L
**Lifestyle interventions** (incorporating physical activity, **dietary change** and behavioral components) should be considered for service users who are experiencing **weight gain** on antipsychotic medications	4	S	H
**Lifestyle interventions** (incorporating **physical activity**, dietary change and behavioral components) should be considered for service users who are experiencing **weight gain** on antipsychotic medications	6	S	H
**Behavioral interventions**			
Lifestyle interventions (incorporating physical activity, dietary change and **behavioral components**) should be considered for service users who are experiencing **weight gain** on antipsychotic medications.	6	S	H
**i) Speech, language, and communication**			
**Assessment of communication needs**			
Offer people a comprehensive biopsychosocial needs assessment by a multidisciplinary team within 4 weeks of entering the rehabilitation service. Include the following as part of the **comprehensive assessment:** current cognitive function, including any **communication needs**	5	S	U.K.
**Provision and training in the use of alternative and augmentative communication systems**			
# When communicating with children and young people with psychosis or schizophrenia and their parents or carers: · take into account the child or young person's developmental level, emotional maturity and cognitive capacity including any learning disabilities, sight or hearing problems or delays in language development · use plain language where possible and clearly explain any clinical language · check that the child or young person and their parents or carers understand what is being said · use **communication aids (such as pictures, symbols, large print, braille, different languages or** sign language) if needed	4	S	E.O.
**j) Sexual functions and intimate relationships**			
**Assessment of sexual functions and intimate relationships**			
# We recommend that in addition to the principles applied in adult patients, side-effect monitoring in children and adolescents receiving antipsychotics takes into account the specific characteristics of this age group. These include: · Effect of elevated prolactin levels on sexual development	1		E.O.
The initial physical health check in the **comprehensive assessment by the rehabilitation service should include sexual health**	5	S	U.K.
Offer people in rehabilitation services a routine physical health check at least annually. The annual physical health check should include **sexual health**	5	S	M
**Education and advice on sexual health**			
Give people clear and accessible **information about** any health risks related to their lifestyle, including: ... ·**sexual and reproductive health**	5	S	L
All women with childbearing potential who take psychotropic medication should be made aware of the potential effects of the medications in pregnancy. The use of reliable **contraceptive methods** should be **discussed**. **Mental health/Emotional functions**	6	C	VL
**k) Mental health/emotional functions**			
**Assessment of mental health**			
# Routinely **monitor** for other **coexisting** mental health problems, including **depression** and anxiety, and substance mis-use, particularly in the early phases of treatment	4	S	E.O.
We suggest that people with schizophrenia are regularly **evaluated** for the presence of **depressive symptoms**. If an instrument is used for evaluation, we suggest the CDSS as the rating instrument of choice	1	C	M
# Routinely **monitor** for other **coexisting** mental health problems, including depression and **anxiety**, and substance mis- use, particularly in the early phases of treatment	4	S	E.O.
**Cognitive behavioral therapy**			
We suggest that people with schizophrenia who have comorbid **depressive symptoms** (with partially remitted psychotic symptoms) are offered **psychosis-specific cognitive behavioral therapy (CBT)** that takes into account the depressive symptoms	1	C	M
**Individual CBTp** should be offered to all individuals diagnosed with schizophrenia whose symptoms have not adequately responded to antipsychotic medication and where persisting symptoms and/or **depression** are being experienced. CBTp can be started during the initial phase, the acute phase or recovery phase including inpatient settings	6	S	H
**Peer support**			
# **Peer-to-peer concepts** may be considered for people with schizophrenia with the aim to achieve greater optimism and recovery. **Psychoeducational approaches** based on the peer-to-peer model may be considered for patients, family members and close confidants to enable alternative paths, have a positive effect on the growth of knowledge and concept of the illness and **reduce subjective distress**	1		E.O.
**Assement of risk of self-harm**			
Offer people a comprehensive biopsychosocial needs assessment by a multidisciplinary team within 4 weeks of entering the rehabilitation service. Include the following as part of the **comprehensive assessment:** · Vulnerabilities, including self-neglect, exploitation and abuse, **and the person's risk of harm to themselves (including suicide) and others**	5	S	U.K.
# We recommend continuously **evaluating suicidal thoughts, plans and behavior**. In particular command hallucinations, persecutory delusions, feelings of alien influence, depressive symptoms and anxiety states should be evaluated as to whether they are having an impact on the incidence of suicidal thoughts or self- harming behavior. We recommend that practitioners strive to avoid akathisia and other debilitating drug side effects and to reduce comorbid substance use	1		E.O.
**l) Mental health/cognitive functions**			
**Assessment of cognitive functions**			
· Offer people a comprehensive biopsychosocial needs assessment by a multidisciplinary team within 4 weeks of entering the rehabilitation service. Include the following as part of the **comprehensive assessment**: ·**developmental history** from birth, including milestones; relationships with peers; and problems at school (**identifying any problems** with social or **cognitive functioning**, motor development and skills or coexisting neurodevelopmental conditions) ·**current cognitive function**, including any communication needs · the person's capacity to give informed consent for their treatment in line with the Mental Capacity Act 2005	5	S	U.K.
**Cognitive remediation therapy**			
**Cognitive remediation therapy** may be considered for individuals diagnosed with schizophrenia who have persisting problems associated with **cognitive difficulties**	6	C	H
We recommend offering **cognitive remediation** to people with schizophrenia who have **impairments in cognitive processes** (attention, learning and memory, executive functions, social cognition or metacognition) to **improve cognitive performance**	1	S	H
# We suggest offering **cognitive remediation** in combination with other psychosocial and rehabilitative treatment methods	1		E.O.
APA suggests that patients with schizophrenia receive **cognitive remediation**	2	C	L
**Assessment of sleep disturbances**			
# If sleep disorders occur in people with schizophrenia, we recommend clarifying and, if possible, treating the cause (e.g. adverse events, obstructive sleep apnea)	1		E.O.
**Education and advice on self-management of sleep**			
Consider providing **advice and support** for good **sleep hygiene** and maximize opportunities for **healthy sleep**. For example, for inpatients, avoid barriers to sleep such as environmental factors or intrusive night-time checks	5	C	L
**Environmental modification**			
Consider providing advice and support for good sleep hygiene and maximize opportunities for **healthy sleep**. For example, for inpatients, avoid barriers to sleep such as **environmental factors** or intrusive night-time checks	5	C	L
**Art therapy**			
# If arts therapies are considered, they should be provided by Health Professions Council (HPC) registered arts therapists, with experience of working with children and young people with psychosis or schizophrenia. The intervention should be provided in groups unless difficulties with acceptability and access and engagement indicate otherwise. Arts therapies should combine psychotherapeutic techniques with activity aimed at promoting creative expression, which is often unstructured and led by the child or young person. Aims of **arts therapies** should include: ·**enabling** children and young people with psychosis or schizophrenia to **experience themselves** differently and to develop new ways of relating to others · helping children and young people to express themselves and to organize their experience into a satisfying aesthetic form · helping children and young people to accept and understand feelings that may have emerged during the creative process (including, in some cases, how they came to have these feelings) at a pace suited to them	4	C	E.O.
**m) Symptoms of psychosis**			
**Assessment of symptoms of psychosis**			
Offer people a comprehensive biopsychosocial needs assessment by a multidisciplinary team within 4 weeks of entering the rehabilitation service. Include the following as part of the comprehensive assessment: · systematic assessment of primary and coexisting mental health problems	5	S	U.K.
**Family interventions (for prevention of relapse and worsening of symptoms)**			
**To reduce** the rate of **recurrence** and **hospitalization**, we recommend offering families of people with a first psychotic episode a **psychotherapeutic family intervention** specifically targeted at first episodes	1	S	H
APA suggests that patients with schizophrenia who have ongoing contact with family receive **family interventions**	2	C	M
# Offer **family intervention** to all families of people with psychosis or schizophrenia who live with or are in close contact with the service user. This can be started either during the acute phase or later, including in inpatient settings	3	S	E.O.
Offer **family intervention** to families of children and young people with psychosis or schizophrenia to **promote recovery**	4	S	L
Continue to offer people with complex psychosis individual cognitive behavioral therapy (CBT) and **family intervention** as recommended by the NICE guideline on psychosis and schizophrenia in adults	5	S	M
· Consider additional **psychological interventions**, especially for people who are not ready to engage in CBT. Use psychological assessment and formulation to identify the most appropriate therapeutic intervention, guided by the person's preferences. Interventions could include: · those focusing on learned behaviors and how context influences behavior · mindfulness approaches where people can be supported to focus on and attend to present experiences · approaches that include a **focus on** wider systems such as **families** or ward environments and their impact on the person	5	C	M
· # **Family intervention** may be particularly useful for families of people with psychosis or schizophrenia who have: · recently relapsed or are at risk of relapse · persisting symptoms	3	C	E.O.
Consider **family intervention** particularly for families of children and young people with psychosis or schizophrenia who have: · recently relapsed or are at risk of relapse · persisting symptoms	4	C	L
# For people with schizophrenia whose illness has not responded adequately to pharmacological or psychological treatment: · Review the diagnosis · Establish that there has been adherence to antipsychotic medication, prescribed at an adequate dose and for the correct duration · Review engagement with and use of psychological treatments and ensure that these have been offered according to this guideline. If family intervention has been undertaken suggest CBT; if CBT has been undertaken suggest **family intervention** for people in close contact with their families	3	S	E.O.
# For children and young people with psychosis or schizophrenia whose illness has not responded adequately to pharmacological or psychological interventions: · Review the diagnosis · Establish that there has been adherence to antipsychotic medication, prescribed at an adequate dose and for the correct duration · Review engagement with and use of psychological interventions and ensure that these have been offered according to this guideline; if family intervention has been undertaken suggest CBT; if CBT has been undertaken suggest **family intervention** for children and young people in close contact with their families	4	S	E.O.
**Family intervention** should be offered to all individuals diagnosed with schizophrenia who are in close contact with or live with family members and should be considered a priority where there are **persistent symptoms** or a **high risk of relapse**. Ten sessions over a three-month period should be considered the minimum effective dose. **family intervention** should **encompass: communication skills, problem solving and psychoeducation**	6	S	H
We suggest performing **psychotherapy and involving the family**, as follows: · Both the service user and the family members ought to be involved · The psychotherapeutic treatment ought to last 3 months to 1 year · It ought to include at least 10 planned sessions · The family's preference for single-family treatment or group psychotherapy with several families ought to be taken into consideration · The relationship between the family members and service user ought to be taken into consideration · The psychotherapy ought to have a specific, supportive, **psychoeducational and therapeutic orientation and include problem-solving training** and the development of a crisis plan	1	C	M
**Mindfulness based approaches**			
Consider additional **psychological interventions**, especially for people who are not ready to engage in CBT. Use psychological assessment and formulation to identify the most appropriate therapeutic intervention, guided by the person's preferences. Interventions could include: · those focusing on learned behaviors and how context influences behavior ·**mindfulness approaches** where people can be supported to **focus on and attend to present experiences** · approaches that include a focus on wider systems such as families or ward environments and their impact on the person	5	C	M
**Meta cognitive training**			
We suggest offering **metacognitive training** to reduce **positive symptoms**	1	C	M
**Cognitive behavioral therapy**			
# Offer **CBT** to all people with psychosis or schizophrenia (delivered as described in recommendation 14.3.7.1). This can be started either during the acute phase or later, including in inpatient settings	3	S	E.O.
Offer **CBT** (delivered as set out in recommendation 9.3.1.28) to all children and young people with psychosis or schizophrenia, particularly for **symptom reduction**. This can be started either during the acute phase or later, including in inpatient settings	4	S	L
Continue to offer people with complex psychosis individual **cognitive behavioral therapy (CBT)** and family intervention as recommended by the NICE guideline on psychosis and schizophrenia in adults	5	S	M
We recommend offering people with schizophrenia **cognitive behavioral therapy**	1	S	H
We suggest also offering **cognitive behavioral therapy** to reduce **psychotic symptoms** in patients who refuse treatment with antipsychotics	1	C	M
# Offer **CBT** to assist in **overy** in people with persisting positive and negative symptoms and for people in remission. Deliver CBT as described in recommendation 14.3.7.1	3	S	E.O.
Offer **CBT** to assist in **promoting recovery** in children and young people with persisting positive and negative symptoms and for those in remission. Deliver CBT as described in recommendation 9.3.1.28	4	S	L
# For people with schizophrenia whose illness has not responded adequately to pharmacological or psychological treatment: · Review the diagnosis · Establish that there has been adherence to antipsychotic medication, prescribed at an adequate dose and for the correct duration · Review engagement with and use of psychological treatments and ensure that these have been offered according to this guideline. If family intervention has been undertaken suggest **CBT**; if CBT has been undertaken suggest family intervention for people in close contact with their families	3	S	E.O.
# For children and young people with psychosis or schizophrenia whose illness has not responded adequately to pharmacological or psychological interventions: · review the diagnosis · establish that there has been adherence to antipsychotic medication, prescribed at an adequate dose and for the correct duration · review engagement with and use of psychological interventions and ensure that these have been offered according to this guideline; if family intervention has been undertaken suggest **CBT**; if CBT has been undertaken suggest family intervention for children and young people in close contact with their families	4	S	E.O.
APA recommends that patients with schizophrenia be treated with **cognitive-behavioral therapy for psychosis (CBTp**)	2	S	M
Individual **CBTp** should be offered to all individuals diagnosed with schizophrenia whose **symptoms** have not adequately responded to antipsychotic medication and where persisting symptoms and/or depression are being experienced. CBTp can be started during the initial phase, the acute phase or recovery phase including inpatient settings	6	S	H
**Other psychological approaches**			
APA suggests that patients with schizophrenia be treated with **supportive psychotherapy**	2	C	L
# **Do not** routinely **offer** counseling and **supportive psychotherapy** (as specific interventions) to children and young people with psychosis or schizophrenia. However, take the child or young person's and their parents' or carers' preferences into account, especially if other more efficacious psychological interventions, such as CBT, family intervention and arts therapies, are not available locally	4	S	E.O.
Consider additional **psychological interventions**, especially for people who are not ready to engage in CBT. Use psychological assessment and formulation to identify the most appropriate therapeutic intervention, guided by the person's preferences. Interventions could include: · those **focusing on learned behaviors** and how **context influences behavior** · mindfulness approaches where people can be supported to focus on and attend to present experiences · approaches that include a focus on wider systems such as families or ward environments and their impact on the person	5	C	M
To improve **psychopathological symptoms**, we suggest offering **music therapy**, art therapy and drama therapy to people with schizophrenia as part of an overall treatment plan and in accordance with the patient's individual needs and preferences	1	C	M
To improve **psychopathological symptoms**, we suggest offering music therapy, **art therapy** and drama therapy to people with schizophrenia as part of an overall treatment plan and in accordance with the patient's individual needs and preferences	1	C	M
To improve **psychopathological symptoms**, we suggest offering music therapy, art therapy and **drama therapy** to people with schizophrenia as part of an overall treatment plan and in accordance with the patient's individual needs and preferences	1	C	M
Group based **art therapy should not be routinely offered** to individuals diagnosed with schizophrenia	6	C	H
**Occupational therapy (ergotherapy)** interventions may be considered as part of an overall treatment plan and in accordance with the patient's individual needs and preferences	1	C	VL
# Consider offering **arts therapies** to all people with schizophrenia, particularly for the alleviation of **negative symptoms**. This can be started either during the acute phase or later, including in inpatient settings	3	C	E.O.
# Consider offering **arts therapies** to assist in promoting recovery, particularly in people with **negative symptoms**	3	C	E.O.
# Consider **arts** to assist in promoting recovery, particularly in children and young people with **negative symptoms**	4	C	E.O.
# Consider **arts therapies** (e.g, dance movement, drama, music or art therapy) for all children and young people with psychosis or schizophrenia, particularly for the alleviation of **negative symptoms**. This can be started either during the acute phase or later, including in inpatient settings	4	C	E.O.
**Physical exercise training**			
# We suggest offering **exercise interventions** (in particular **aerobic endurance training**, yoga), taking into account the patient's physical abilities	1		E.O.
We suggest offering **movement interventions** to people with schizophrenia as part of a multimodal overall therapy concept, depending on people's symptoms and preferences and taking into account their physical abilities	1	C	M

The QoE and SoR are classified following the harmonized classification in the Supplementary Table. The highest available evidence available for each recommendation has been assigned as QoE. QoE, quality of evidence; H, high; M, moderate; L, low; VL, very low; E.O., experts' opinion; U.K., United Kingdom prevalence studies, GRADE methodology was not used. Ref, reference guideline; Ref 1, DGPPN-S3; REF 2, APA; REF 3, NICE-1; REF 4, NICE-2; REF5, NICE-3; REF6, SIGN; SoR, strength of recommendation; S, strong; C, conditional; #, recommendation based on experts' opinion only. Strong recommendations based on high quality evidence are framed with a thick border.

For assessment recommendations, “Mental/Emotional functions” and “Education and Vocation” were the functioning domains with the highest number of recommendations. Assessment recommendations for “Interpersonal interactions and relations,” “Exercise and fitness,” and “Speech, language and communication” were provided in NICE-3, and “Activities of daily living” in NICE-1 and NICE-3. In terms of providing specific assessment recommendations, “self-management” or “community and social life” domains were not addressed in the CPG.

Regarding intervention recommendations, all six CPG provided many recommendations addressing “symptoms of psychosis” which was the domain with the highest number of recommendations. A high number of interventions were also provided for “Education and Vocation” and “Self-management,” 12 and nine, respectively. All CPG, except for SIGN and NICE-1 respectively, addressed “Education and Vocation” as well as “Mental/cognitive functions” and “Interpersonal interactions and relations.” A total of six domains, out of 13 were targeted by low number of recommendations (4 or less) including “community and social life” (addressed by NICE-3), and “activities of daily living” (addressed by NICE-3 and DGPPN).

The QoE for given recommendations was high for 20.77% (*n* = 27) of the recommendations. The same number of recommendations had a moderate QoE (*n* = 27; 20.77%) while *n* = 20 (15.38%) had low QoE. Only two recommendations (1.52%) had very low QoE, and nine (6.92%) were based on U.K. prevalence studies. A total *n* = 45 (34.62%) of the recommendations is based on experts' opinions. Experts' opinions are used for recommendations on generally good clinical practice (e.g., informing carers on their statutory rights. See Table 3), as well as to support clinically available interventions in lack of sufficient available evidence (e.g., “We suggest offering cognitive remediation in combination with other psychosocial and rehabilitative treatment methods”). SoR is strong for more than half of the recommendations (*n* = 81; 62.3%). Within these strong recommendations, *n* = 25 is based on Experts' opinion. A minority of recommendations (*n* = 37; 28.46%) is conditional, while the rest (*n* = 12; 9.23%) indicates no strength of recommendation and is based on Experts' opinion.

## 4. Discussion

In this systematic review we summarized existing recommendations from high-quality CPG on rehabilitation interventions for people with schizophrenia. Only one CPG with a specific focus on the rehabilitation of schizophrenia was found. This suggests a gap in current CPG production process, with a relative lack of information on this topic. This seems particularly relevant in view of the recent publication of the WHO World Mental Health Report (WMHR), which urges nations and professionals to work toward empowering people to regain independence and live a life in full autonomy through the implementation of recovery-oriented interventions, most of which may be categorized as rehabilitation interventions (16).

Despite the scarcity of CPG on rehabilitation of schizophrenia, we identified general CPG that included several recommendations that are, either directly or indirectly, relevant to the assessment of rehabilitation needs and to the provision of rehabilitation interventions. To our knowledge, this is the first systematic overview of recommendations in this important field of intervention, which will be used to inform the WHO PIR and, hopefully, it may also be of interest to countries and professionals aiming to fully implement the concept of rehabilitation, in line with the WHO WMHR.

In terms of quality of available evidence, only a minority of target domains and recommendations are supported by high-quality evidence. Notably, expert opinions often supported recommendations on good clinical practice as well as on specific interventions, even in absence of a supporting background body of evidence. This applies, for example, to recommendations on physical activity, or those on the combination of individual and family interventions, or those in favor of supported employment. Despite this relatively low quality of background evidence, the strength of many of these recommendations was *strong*. This is in line with current standards of CPG development, as in addition to the evidence base, other considerations, such as for example values, preferences, feasibility and equity considerations should also be considered in drafting recommendations and their strength. We note, therefore, that in the field of rehabilitation there is a discrepancy between the scarcity of data on one side, and the high value attributed to rehabilitation interventions by professionals and by a variety of different stakeholders, including people with living experience of mental health conditions, on the other side. Increasing research and knowledge about this complex subject could increase clarity on what the key elements of rehabilitation in schizophrenia are. Research in this field however is hard to conduct due to long follow-up times, heterogeneous settings, complex outcomes, and lack of resources.

Looking at the total number of recommendations on specific topics (functioning domains, Table 2), some areas are not addressed adequately by available CPG, for example “Activity of daily living,” “Interpersonal interactions and relations,” “Community and social life,” and “Carer and family support.” Conversely, we included 16 recommendations addressing “symptoms of psychosis.” This disbalance is probably related to the inconsistent definition of rehabilitation in this field, and to an increased difficulty in effectively research outcomes such as “community involvement and social life” (in opposition to the reduction of psychotic symptoms).

The concept of rehabilitation is tightly related to the concept of recovery which, in the field of mental health, gained prominence after the implementation of deinstitutionalization with ever greater relevance of community services and interventions to help people achieve recovery. Recovery from a mental illness has been described as “*a deeply personal, unique process of changing one's attitudes, values, feelings, goals, skills, and/or roles. It is a way of living a satisfying, hopeful, and contributing life even with limitations caused by illness. Recovery involves the development of new meaning and purpose in one's life as one grows beyond the catastrophic effects of mental illness*” (13). A more recent definition has been formulated by merging the professionals' most commonly cited themes describing the meaning of the term rehabilitation which reads: “*A whole system approach to recovery from mental ill health which maximizes an individual's quality of life and social inclusion by encouraging their skills, promoting independence and autonomy in order to give them hope for the future and which leads to successful community living through appropriate support*” (36). Ultimately, in accordance with the WHO WMHR, if recovery from a mental disorder is the process of regaining a better mental health, rehabilitation is the body of procedures helping the individual in this process and fostering the ability to connect, function, cope and thrive (16). Further promoting a switch of paradigm from a “hospital/asylum-centric” approach to a community-centered care at a policy-making level, will stimulate the research and implementation of rehabilitation techniques focusing on recovery rather than pure reduction of symptoms (15). Still, it has to be considered that schizophrenia is a lifelong condition characterized by prodromal and residual subclinical disturbances, having implications in chronic disability (37, 38), with a 3.5%−11% per month risk of abrupt relapse to the pre-treatment condition for treatment adherent and non-adherent people in treatment for psychoses respectively (39, 40). Therefore, the long-term management and reduction of key symptoms have to be taken into account in the rehabilitation of schizophrenia as one of the factors involved in long-term recovery.

This review needs to be interpreted in the light of some limitations. First, CPG should be considered with a recognition of their inherent limitations. These recommendations are formulated based on the currently available evidence and do not encompass all potential actions required to mobilize community resources and opportunities, strengthen social cohesion and social capital, and overall integrate formal and informal care services while considering the local context. Nonetheless, the absence of these considerations does not imply that such actions should be excluded from intervention programs. All parties involved should acknowledge the importance of tailoring rehabilitation projects to accommodate individual, familial, and cultural contexts, as well as the location of service delivery, in order to ensure effective implementation. Clinicians are therefore urged to make a concerted effort to integrate evidence-based CPG with the specific needs of individuals who have experienced mental disorders. By doing so, they can provide guidance that empowers individuals to utilize resources, tools, and opportunities that align with their personal aspirations, as well as their social and clinical circumstances. A second limitation is inherent to the systematic review methodology itself, due to the potential exclusion of titles, and therefore potentially relevant information, not emerging from the literature search. In this regard, the strings used for this research were developed on the basis of the WHO indication on the systematic search of literature for the development of the PIR for all conditions. Another limitation arises from the sole inclusion of English language CPG, which was driven by feasibility considerations. We recognize that the omission of CPG in other languages could pose a significant constraint, as the outcome of the current review may predominantly reflect Western practices. Consequently, contexts in which alternative approaches, involving for example non-specialist community workers, volunteers, activists, and peers, play pivotal roles may not have received sufficient visibility.

## 5. Conclusion

In conclusion, six CPG based on available evidence, relevant WHO guidelines and expert opinion will inform the development of the WHO PIR for schizophrenia and related psychosis. This review revealed an imbalance in the domains addressed and a lack of indications in poor-resource settings. Priority targets and interventions in the rehabilitation in schizophrenia are still unclear in terms of significance and feasibility.

The scientific community is called to embrace the challenge posed by the WHO WMHR 2022 (16). To promote a more thorough and holistic approach to rehabilitation in this field, it will be important to research on and implement pragmatic solutions in somewhat neglected areas of the rehabilitation of schizophrenia such as “Community and social life.” Within the WMHR logic, supporting people in accessing health and social services, such as psychosocial rehabilitation, housing, or welfare benefits, are all crucial aspects of care. All these actions ought to be carried out with the intention of fostering community involvement, engagement, and legal ability. The WHO PIR will help face this important challenge in the field of schizophrenia.

## Author contributions

Conceptualization: RS, YE, CB, and LT. Methodology: YE, MR, CB, and LT. Software: RS and YE. Formal analysis: RS. Investigation: RS, YE, and MR. Resources: CB and LT. Data curation: RS, YE, MR, and LT. Writing—original draft preparation: RS and LT. Writing—review and editing: CB, YE, and MR. Project administration: YE, CB, and LT. All authors have read and agreed to the published version of the manuscript.
