# Supplementary material for: A systematic review of Clinical Practice Guidelines for the development of the WHO's Package of Interventions for Rehabilitation: focus on schizophrenia
Source: Front Public Health. 2023 Aug 15;11:1215617. doi: 10.3389/fpubh.2023.1215617 (PMC10465692; doi:10.3389/fpubh.2023.1215617)
Supplement: Supplementary file 1 [file Table_1.docx]

| **Supplementary Table 1** Abstract and full text screening of files for inclusion in the review. | | | | | | | | | | | | | | | | | | | | | | | | | | | |
| --- | --- | --- | --- | --- | --- | --- | --- | --- | --- | --- | --- | --- | --- | --- | --- | --- | --- | --- | --- | --- | --- | --- | --- | --- | --- | --- | --- |
|  | | | **ABSTRACT SCREENING** | | | | | | | | | | | **FULL TENT SCREENING** | | | | | | | | | | | | | |
|  |  |  | **First Reviewer** | | | | | **Second Reviewer** | | | | |  | **First Reviewer** | | | | | | **Second Reviewer** | | | | | | **Final decision** | |
| 1st Author / Editors | **year** | **Title** | **C1** | **C2** | **C3** | **C4** | **T o t a l** | **C1** | **C2** | **C3** | **C4** | **T o t a l** | **I n c l**  **u s i o n** | **C1** | **C2** | **C3** | **C5** | **C6** | **T o t a l** | **C1** | **C2** | **C3** | **C5** | **C6** | **T o t a l** | **I n c l u s i o n** | **C o m m e n t s** |
| Addington, D. | 2017 | Canadian Guidelines for the Assessment and Diagnosis of Patients with Schizophrenia Spectrum and Other Psychotic Disorders | Y | Y | Y | Y | **Y** | Y | Y | N | Y | **N** | **N** |  |  |  |  |  |  |  |  |  |  |  |  |  |  |
| AHQAC (es) agency for health quality and assessment of catalonia |  | Guía de práctica clínica sobre la esquizofrenia y el trastorno psicótico incipiente | N | N | N | N | **N** | N |  |  |  | **N** | **N** |  |  |  |  |  |  |  |  |  |  |  |  |  |  |
| AHQAC (es) agency for health quality and assessment of catalonia |  | Comparación de la eficacia de las formas farmacéuticas de liberación retardada (depot) versus las formas farmacéuticas orales de los antipsicóticos (típicos y atípicos), comercializados en Cataluña, en pacientes con un diagnóstico de esquizofrenia | N | N | N | N | **N** | N |  |  |  | **N** | **N** |  |  |  |  |  |  |  |  |  |  |  |  |  |  |
| Ayala, Lester | 2018 | Is a guideline based treatment cost-effective for a middle income country? Results from a study with Mexican adolescents | N | N | N | N | **N** | N |  |  |  | **N** | **N** |  |  |  |  |  |  |  |  |  |  |  |  |  |  |
| Bastiampillai, T. | 2016 | NICE guidelines for schizophrenia: can art therapy be justified? | N | N | N | N | **N** | N |  |  |  | **N** | **N** |  |  |  |  |  |  |  |  |  |  |  |  |  |  |
| Bollini, P. | 2008 | Indicators of conformance with guidelines of schizophrenia treatment in mental health services | N | N | N | N | **N** | N |  |  |  | **N** | **N** |  |  |  |  |  |  |  |  |  |  |  |  |  |  |
| Bradshaw, T. | 2012 | Developing a healthy living intervention for people with early psychosis using the Medical Research Council's guidelines on complex interventions: Phase 1 of the HELPER – InterACT programme | Y | N | N | N | **N** | Y | N | Y | Y | **N** | **N** |  |  |  |  |  |  |  |  |  |  |  |  |  |  |
| Buchanan, Robert W. | 2010 | The 2009 schizophrenia PORT psychopharmacological treatment recommendations and summary statements | Y | Y | N | N | **N** | Y | Y | N | Y | **N** | **N** |  |  |  |  |  |  |  |  |  |  |  |  |  |  |
| Castelein, S. | 2013 | Dutch guideline on Schizophrenia 2012: Basic care within the areas of psychosocial interventions and nursing care | Y | Y | Y | Y | **Y** | Y | Y | Y | Y | **Y** | **Y** |  |  |  |  |  | **N** |  |  |  |  |  | **N** | **N** | **Not in english** |
| Castelein, S. | 2013 | [Dutch guideline on Schizophrenia 2012: basic care within the areas of psychosocial interventions and nursing care] | Y | Y | Y | Y | **Y** | Y | Y | Y | Y | **Y** | **Y** | Y | Y | N | Y | Y | **N** | Y | Y | N | Y | Y | **N** | **N** |  |
| Castle, D. J. | 2017 | The 2016 Royal Australian and New Zealand College of Psychiatrists guidelines for the management of schizophrenia and related disorders | Y | Y | Y | Y | **Y** | Y | Y | Y | Y | **Y** | **Y** | Y | Y | Y | Y | Y | **N** | Y | Y | Y | Y | Y | **N** | **N** |  |
| Chudzynski, Joy | 2009 | A guide to treating severe mental illness with cognitive behavioral therapy | N | N | N | N | **N** | N |  |  |  | **N** | **N** |  |  |  |  |  |  |  |  |  |  |  |  |  |  |
| Claxton, Melanie | 2017 | Do family interventions improve outcomes in early psychosis? A systematic review and meta-analysis | N | N | N | N | **N** | N |  |  |  | **N** | **N** |  |  |  |  |  |  |  |  |  |  |  |  |  |  |
| Crockford, David | 2017 | Canadian schizophrenia guidelines: Schizophrenia and other psychotic disorders with coexisting substance use disorders | Y | Y | Y | Y | **Y** | Y | Y | Y | Y | **Y** | **Y** | Y | Y | Y | Y | Y | **N** | Y | Y | Y | Y | Y | **N** | **N** | **Adaptation of other guideline** |
| de Jesus Mari, Jair | 2009 | Packages of care for schizophrenia in low-and middle-income countries | Y | Y | Y | Y | **Y** | Y | Y | Y | Y | **N** | **N** |  |  |  |  |  |  |  |  |  |  |  |  |  |  |
| Dixon, L. B. | 2010 | The 2009 schizophrenia PORT psychosocial treatment recommendations and summary statements | Y | Y | Y | Y | **Y** | Y | Y | Y | Y | **Y** | **Y** | N | Y | Y | Y | N | **N** | N | Y | Y | Y | N | **N** | **N** |  |
| Dixon, Lisa | 2009 | Guideline watch (September 2009): practice guideline for the treatment of patients with schizophrenia | Y | Y | Y | Y | **Y** | Y | Y | N | N | **N** | **N** |  |  |  |  |  |  |  |  |  |  |  |  |  |  |
| Dixon, Lisa B. | 2010 | The 2009 schizophrenia PORT psychosocial treatment recommendations and summary statements | Y | Y | Y | Y | **Y** | Y | Y | Y | Y | **Y** | **Y** | Y | Y | Y | N | N | **N** | Y | Y | Y | N | N | **N** | **N** |  |
| Dondé, C. | 2018 | Management of depression in patients with schizophrenia spectrum disorders: A critical review of international guidelines | N | N | N | N | **N** | N |  |  |  | **N** | **N** |  |  |  |  |  |  |  |  |  |  |  |  |  |  |
| French, Paul | 2010 | Promoting recovery in early psychosis: A practice manual | N | N | N | N | **N** | N |  |  |  | **N** | **N** |  |  |  |  |  |  |  |  |  |  |  |  |  |  |
| Gaebel, W. | 2011 | Schizophrenia guidelines across the world: a selective review and comparison | N | N | N | N | **N** | N |  |  |  | **N** | **N** |  |  |  |  |  |  |  |  |  |  |  |  |  |  |
| Galletly, Cherrie | 2016 | Royal Australian and New Zealand College of Psychiatrists clinical practice guidelines for the management of schizophrenia and related disorders | Y | Y | Y | Y | **Y** | Y | Y | Y | Y | **Y** | **Y** | Y | Y | Y | Y | Y | **Y** | Y | Y | Y | Y | Y | **Y** | **Y** |  |
| Gomez-Restrepo, C. | 2014 | [Clinical Practice Guidelines for Diagnosis, Treatment and Beginning of Psychosocial Rehabilitation of Adults With Schizophrenia: "Do Well the Things That do Well"] | N | N | N | N | **N** | N |  |  |  | **N** | **N** |  |  |  |  |  |  |  |  |  |  |  |  |  |  |
| Granholm, Eric L. | 2016 | Cognitive-behavioral social skills training for schizophrenia: A practical treatment guide | N | N | N | N | **N** | N |  |  |  | **N** | **N** |  |  |  |  |  |  |  |  |  |  |  |  |  |  |
| Grover, Sandeep | 2017 | Clinical practice guidelines for management of schizophrenia | Y | Y | Y | Y | **Y** | Y | Y | Y | Y | **Y** | **Y** | Y | Y | Y | N | N | **N** | Y | Y | Y | N | N | **N** | **N** |  |
| Gühne, Uta | 2015 | S3 guideline on psychosocial therapies in severe mental illness: evidence and recommendations | Y | Y | Y | Y | **Y** | Y | Y | Y | Y | **Y** | **Y** | Y | N | Y | Y | Y | **N** | Y | Y | Y | Y | Y | **Y** | **N** |  |
| Hadjulis, M. | 2018 | Clinical guidelines for the management of schizophrenia: Pharmacological and psychological interventions (III) | Y | Y | Y | Y | **Y** | Y | Y | Y | Y | **Y** | **Y** |  |  |  |  |  | **N** |  |  |  |  |  | **N** | **N** | **Not in english** |
| HAS (FR) French national authority for health |  | Dangerosité psychiatrique: étude et évaluation des facteurs de risque de violence hétéro-agressive chez les personnes ayant des troubles schizophréniques ou des troubles de l'humeur. Audition publique | N | N | N | N | **N** | N |  |  |  | **N** | **N** |  |  |  |  |  |  |  |  |  |  |  |  |  |  |
| Hasan, A. | 2015 | Update of the DGPPN S3-guidelines for the treatment of schizophrenia: Current status | Y | Y | Y | Y | **Y** | Y | Y | Y | Y | **Y** | **Y** | Y | Y | Y | Y | Y | **Y** | Y | Y | Y | Y | Y | **Y** | **Y** |  |
| Hasan, A. | 2020 | [Revised S3 guidelines on schizophrenia : Developmental process and selected recommendations] | Y | Y | Y | Y | **Y** | Y | Y | Y | Y | **Y** | **Y** | Y | Y | Y | Y | Y | **N** | Y | Y | Y | Y | Y | **Y** | **N** | **Not in english** |
| Hasan, Alkomiet | 2013 | World Federation of Societies of Biological Psychiatry (WFSBP) guidelines for biological treatment of schizophrenia, part 2: update 2012 on the long-term treatment of schizophrenia and management of antipsychotic-induced side effects | Y | Y | Y | Y | **Y** | Y | Y | N | Y | **N** | **N** |  |  |  |  |  |  |  |  |  |  |  |  |  |  |
| HTA DOH (MY) HTA unit, ministery of health, malaysia |  | Management of Schizophrenia in Adults | Y | Y | Y | Y | **Y** | Y | Y | Y | Y | **Y** | **Y** | Y | Y | Y | N | N | **N** | Y | Y | N | N | N | **N** | **N** |  |
| IACS (es) Guìasalud-Aragon institute of health sciences |  | Guía de Práctica Clínica de Intervenciones Psicosociales en el Trastorno Mental Grave | N | N | N | N | **N** | N |  |  |  | **N** | **N** |  |  |  |  |  |  |  |  |  |  |  |  |  |  |
| IQWIG (DE) institute for quality and efficiency in health care |  | Lurasidon: Nutzenbewertung gemäß § 35a SGB V | N | N | N | N | **N** | N |  |  |  | **N** | **N** |  |  |  |  |  |  |  |  |  |  |  |  |  |  |
| Jablensky, Assen | 2017 | The 2016 RANZCP guidelines for the management of schizophrenia and related disorders—What’s next? | N | N | N | N | **N** | N |  |  |  | **N** | **N** |  |  |  |  |  |  |  |  |  |  |  |  |  |  |
| Janssen, B. | 2010 | Improving outpatient treatment in schizophrenia: Effects of computerized guideline implementation—Results of a multicenter-study within the German Research Network on Schizophrenia | N | N | N | N | **N** | N |  |  |  | **N** | **N** |  |  |  |  |  |  |  |  |  |  |  |  |  |  |
| JBI (AU) joanna briggs institute |  | The perception and experience of stigma among people with schizophrenia (Best Practice 16(4)) | N | N | N | N | **N** | N |  |  |  | **N** | **N** |  |  |  |  |  |  |  |  |  |  |  |  |  |  |
| JBI (AU) joanna briggs institute |  | Complex interactive factors which determine carers' experiences of seeking help for relatives with first- episode psychosis (Best Practice 18(10)) | N | N | N | N | **N** | N |  |  |  | **N** | **N** |  |  |  |  |  |  |  |  |  |  |  |  |  |  |
| JBI (AU) joanna briggs institute |  | Caregiving experiences of families living with persons with schizophrenia (Best Practice 18(6)) | N | N | N | N | **N** | N |  |  |  | **N** | **N** |  |  |  |  |  |  |  |  |  |  |  |  |  |  |
| Kealey, E. | 2014 | Quality concerns in antipsychotic prescribing for youth: a review of treatment guidelines | N | N | N | N | **N** | N |  |  |  | **N** | **N** |  |  |  |  |  |  |  |  |  |  |  |  |  |  |
| Keller, William R. | 2014 | Community adherence to schizophrenia treatment and safety monitoring guidelines | N | N | N | N | **N** | N |  |  |  | **N** | **N** |  |  |  |  |  |  |  |  |  |  |  |  |  |  |
| Kendall, Tim | 2016 | NICE v SIGN on psychosis and schizophrenia: Same roots, similar guidelines, different interpretations | N | N | N | N | **N** | N |  |  |  | **N** | **N** |  |  |  |  |  |  |  |  |  |  |  |  |  |  |
| Killackey, Eóin | 2008 | Psychosocial interventions in clinical practice guidelines for schizophrenia | N | N | N | N | **N** | N |  |  |  | **N** | **N** |  |  |  |  |  |  |  |  |  |  |  |  |  |  |
| Kuipers, Elizabeth | 2014 | Management of psychosis and schizophrenia in adults: summary of updated NICE guidance | Y | Y | Y | Y | **Y** | Y | Y | Y | Y | **Y** | **Y** | N | Y | Y | Y | Y | **N** | N | Y | Y | Y | Y | **N** | **N** |  |
| Lahera, Guillermo | 2018 | Functional recovery in patients with schizophrenia: recommendations from a panel of experts | Y | Y | Y | Y | **Y** | Y | Y | Y | Y | **Y** | **Y** | N | Y | Y | Y | N | **N** | N | Y | Y | Y | N | **N** | **N** |  |
| Le Boutillier, Clair | 2011 | What does recovery mean in practice? A qualitative analysis of international recovery-oriented practice guidance | N | N | N | N | **N** | N |  |  |  | **N** | **N** |  |  |  |  |  |  |  |  |  |  |  |  |  |  |
| Lecomte, Tania | 2017 | Canadian treatment guidelines on psychosocial treatment of schizophrenia in children and youth | Y | Y | Y | Y | **Y** | Y | Y | Y | Y | **Y** | **Y** | Y | Y | Y | Y | Y | **N** | Y | Y | Y | Y | Y | **N** | **N** | **Adaptation of other guidelines** |
| Margariti, M. | 2018 | Clinical guidelines for the management of schizophrenia: Aims and limitations (Ι) | Y | Y | Y | Y | **Y** | Y | Y | N | Y | **N** | **N** |  |  |  |  |  |  |  |  |  |  |  |  |  |  |
| Margariti, M. | 2018 | Clinical guidelines for the management of schizophrenia (II): Community service-level interventions and the role of primary care | Y | Y | Y | Y | **Y** | Y | Y | N | Y | **N** | **N** |  |  |  |  |  |  |  |  |  |  |  |  |  |  |
| Markkula, Niina | 2011 | Adherence to guidelines and treatment compliance in the Chilean national program for first-episode schizophrenia | N | N | N | N | **N** | N |  |  |  | **N** | **N** |  |  |  |  |  |  |  |  |  |  |  |  |  |  |
| Mas-Exposito, L. | 2016 | Social cognition interventions for persons with schizophrenia: evidence and clinical practice guidelines | Y | Y | Y | Y | **Y** | Y | Y | Y | Y | **Y** | **Y** | N | Y | Y | Y | N | **N** | N | Y | Y | Y | N | **N** | **N** |  |
| McGorry, Patrick | 2012 | Early intervention for psychosis in Asia | N | N | N | N | **N** | N |  |  |  | **N** | **N** |  |  |  |  |  |  |  |  |  |  |  |  |  |  |
| McKenna, P. J. | 2015 | Selective reporting of results in guidelines | N | N | N | N | **N** | N |  |  |  | **N** | **N** |  |  |  |  |  |  |  |  |  |  |  |  |  |  |
| Meyer, Piper S. | 2010 | A guide to implementation and clinical practice of illness management and recovery for people with schizophrenia | N | N | N | N | **N** | N |  |  |  | **N** | **N** |  |  |  |  |  |  |  |  |  |  |  |  |  |  |
| Muscettola, G. | 2010 | An appraisal of the major: Guidelines on the pharmacotherapy of schizophrenia | N | N | N | N | **N** | N |  |  |  | **N** | **N** |  |  |  |  |  |  |  |  |  |  |  |  |  |  |
| Newton, R. | 2012 | Practical guidelines on the use of paliperidone palmitate in schizophrenia | Y | Y | Y | Y | **Y** | Y | Y | N | Y | **N** | **N** |  |  |  |  |  |  |  |  |  |  |  |  |  |  |
| NICE (UK) National institute for health and care excellence | 2009 | National Institute for Health and Clinical Excellence: Guidance | Y | Y | Y | Y | **Y** | Y | Y | Y | N | **N** | **N** |  |  |  |  |  |  |  |  |  |  |  |  |  |  |
| NICE (UK) National institute for health and care excellence | 2016 | National Institute for Health and Clinical Excellence: Guidance | Y | Y | Y | Y | **Y** | Y | Y | Y | Y | **Y** | **Y** | Y | Y | Y | Y | Y | **Y** | Y | Y | Y | Y | Y | **Y** | **Y** |  |
| NICE (UK) National institute for health and care excellence |  | Psychosis with coexisting substance misuse (CG120) | Y | Y | Y | Y | **Y** | Y | Y | Y | Y | **Y** | **Y** | Y | Y | Y | Y | Y | **N** | Y | Y | Y | Y | Y | **N** | **N** | **Duplicate of other guideline** |
| NICE (UK) National institute for health and care excellence |  | Psychosis and schizophrenia in children and young people: recognition and management (CG155) | Y | Y | Y | Y | **Y** | Y | Y | Y | Y | **Y** | **Y** | Y | Y | Y | Y | Y | **Y** | Y | Y | Y | Y | Y | **Y** | **N** | **Duplicate of other guideline** |
| NICE (UK) National institute for health and care excellence |  | Psychosis and schizophrenia in adults: prevention and management (CG178) | Y | Y | Y | Y | **Y** | Y | Y | Y | Y | **Y** | **Y** | Y | Y | Y | Y | Y | **Y** | Y | Y | Y | Y | Y | **Y** | **Y** |  |
| NICE (UK) National institute for health and care excellence | 2020 | Rehabilitation for adults with complex psychosis | Y | Y | Y | Y | **Y** | Y | Y | Y | Y | **Y** | **Y** | Y | Y | Y | Y | Y | **Y** | Y | Y | Y | Y | Y | **Y** | **Y** |  |
| Norman, Ross | 2017 | Canadian treatment guidelines on psychosocial treatment of schizophrenia in adults | Y | Y | Y | Y | **Y** | Y | Y | Y | Y | **Y** | **Y** | Y | Y | Y | Y | Y | **N** | Y | Y | Y | Y | Y | **N** | **N** | **Adaptation of other guidelines** |
| Orygen | 2016 | Australian clinical guidelines for early psychosis | Y | Y | Y | Y | **Y** | Y | Y | Y | Y | **Y** | **Y** | Y | N | N | N | N | **N** | Y | N | Y | N | N | **N** | **N** |  |
| OSTEBA (es). Basque office for health tecnology assesment |  | Seguimiento grupal de pacientes psicóticos en la Red Pública de salud mental de la Comunidad Autónoma Vasca (Informe n° D-09-06) | N | N | N | N | **N** | N |  |  |  | **N** | **N** |  |  |  |  |  |  |  |  |  |  |  |  |  |  |
| OSTEBA (es). Basque office for health tecnology assesment |  | Revisión de los tratamientos psicológicos en primeros episodios psicóticos (Informe n° E-10-08) | N | N | N | N | **N** | N |  |  |  | **N** | **N** |  |  |  |  |  |  |  |  |  |  |  |  |  |  |
| OSTEBA (es). Basque office for health tecnology assesment |  | Relación entre el consumo de cannabis y otras drogas y patología psiquiátrica en adolescentes. Propuesta de atención en Salud Mental intra y extrahospitalaria de Osakidetza en Euskadi (Informe nº: Osteba D-14-04) | N | N | N | N | **N** | N |  |  |  | **N** | **N** |  |  |  |  |  |  |  |  |  |  |  |  |  |  |
| OSTEBA (es). Basque office for health tecnology assesment |  | Mapa geográfico de salud mental: variación geográfica en psicosis y asociación con factores ambientales (Informe nº: Osteba D-14-02) | N | N | N | N | **N** | N |  |  |  | **N** | **N** |  |  |  |  |  |  |  |  |  |  |  |  |  |  |
| OSTEBA (es). Basque office for health tecnology assesment |  | Evaluación de la efectividad de la intervención psicológica en los primeros episodios psicóticos mediante tratamiento multidisciplinar (Informe nº: Osteba E-14-13) | N | N | N | N | **N** | N |  |  |  | **N** | **N** |  |  |  |  |  |  |  |  |  |  |  |  |  |  |
| OSTEBA (es). Basque office for health tecnology assesment |  | Estrategias terapéuticas en la esquizofrenia refractaria o esquizofrenia resistente a tratamiento (Informe nº: Osteba D/12/04) | N | N | N | N | **N** | N |  |  |  | **N** | **N** |  |  |  |  |  |  |  |  |  |  |  |  |  |  |
| Petrakis, Melissa | 2011 | Fidelity to clinical guidelines using a care pathway in the treatment of first episode psychosis | N | N | N | N | **N** | N |  |  |  | **N** | **N** |  |  |  |  |  |  |  |  |  |  |  |  |  |  |
| Remington, G. | 2017 | Guidelines for the Pharmacotherapy of Schizophrenia in Adults | Y | Y | Y | Y | **Y** | Y | Y | N | Y | **N** | **N** |  |  |  |  |  |  |  |  |  |  |  |  |  |  |
| Saddichha, S. | 2014 | Clinical practice guidelines in psychiatry: more confusion than clarity? A critical review and recommendation of a unified guideline | Y | Y | Y | Y | **Y** | Y | Y | Y | Y | **Y** | **Y** | N | Y | Y | N | N | **N** | N | N | N | N | N | **N** | **N** |  |
| Salokangas, R. K. | 2013 | [Update on current care guidelines: schizophrenia] | Y | Y | Y | Y | **Y** | Y | Y | Y | Y | **Y** | **Y** |  |  |  |  |  | **N** |  |  |  |  |  | **N** | **N** | **Not in english** |
| Sandström, Boel | 2014 | Mapping attitudes and awareness with regard to national guidelines: An e‐mail survey among decision makers | N | N | N | N | **N** | N |  |  |  | **N** | **N** |  |  |  |  |  |  |  |  |  |  |  |  |  |  |
| Sandström, Boel | 2014 | ‘How do we know if this is the best?’ Mental health‐care professionals' views on national guidelines for psychosocial interventions | N | N | N | N | **N** | N |  |  |  | **N** | **N** |  |  |  |  |  |  |  |  |  |  |  |  |  |  |
| Saravane, D. | 2009 | [Drawing up guidelines for the attendance of physical health of patients with severe mental illness] | Y | Y | Y | Y | **Y** | Y | Y | N | N | **N** | **N** |  |  |  |  |  |  |  |  |  |  |  |  |  |  |
| Schmidt, Stefanie Julia | 2015 | EPA guidance on the early intervention in clinical high risk states of psychoses | Y | Y | Y | Y | **Y** | Y | Y | Y | Y | **Y** | **Y** | Y | Y | Y | Y | Y | **Y** | Y | Y | Y | Y | Y | **Y** | **Y** |  |
| SIGN (GB) scottish intercollegiate guidelines network |  | Management of schizophrenia (SIGN CPG 131) | Y | Y | Y | Y | **Y** | Y | Y | Y | Y | **Y** | **Y** | Y | Y | Y | Y | Y | **Y** | Y | Y | Y | Y | Y | **Y** | **Y** |  |
| SIGN (GB) scottish intercollegiate guidelines network |  | Assessment, diagnosis and interventions for autism spectrum disorders (SIGN CPG 145) | N | N | N | N | **N** | N |  |  |  | **N** | **N** |  |  |  |  |  |  |  |  |  |  |  |  |  |  |
| Slade, Mike | 2009 | Personal recovery and mental illness: A guide for mental health professionals | N | N | N | N | **N** | N |  |  |  | **N** | **N** |  |  |  |  |  |  |  |  |  |  |  |  |  |  |
| SST (DK) Danish health authority |  | National Klinisk Retningslinje for behandling af patienter med skizofreni og komplekse behandlingsforløb - vedvarende symptomer, misbrug eller mangelfuld behandlingstilknytning | N | N | N | N | **N** | N |  |  |  | **N** | **N** |  |  |  |  |  |  |  |  |  |  |  |  |  |  |
| Steinert, T. | 2009 | [Update of the British schizophrenia guideline (2009): not light fare, but a milestone] | Y | Y | Y | Y | **Y** | N |  |  |  | **N** | **N** |  |  |  |  |  |  |  |  |  |  |  |  |  |  |
| Stubbs, B. | 2018 | EPA guidance on physical activity as a treatment for severe mental illness: a meta-review of the evidence and Position Statement from the European Psychiatric Association (EPA), supported by the International Organization of Physical Therapists in Mental Health (IOPTMH) | Y | Y | Y | Y | **Y** | Y | Y | Y | Y | **Y** | **Y** | Y | Y | Y | Y | Y | **Y** | Y | Y | Y | Y | Y | **Y** | **Y** |  |
| Szulc, A. | 2019 | Recommendations for the treatment of schizophrenia with negative symptoms. Standards of pharmacotherapy by the Polish Psychiatric Association (Polskie Towarzystwo Psychiatryczne), part 2 | Y | Y | Y | Y | **Y** | Y | Y | N | Y | **N** | **N** |  |  |  |  |  |  |  |  |  |  |  |  |  |  |
| The American Psychiatric Association | 2020 | Practice guideline for treatment of patients with  Schizophrenia | Y | Y | Y | Y | **Y** | Y | Y | Y | Y | **Y** | **Y** | Y | Y | Y | Y | Y | **Y** | Y | Y | Y | Y | Y | **Y** | **Y** |  |
| Thornicroft, Graham | 2010 | WPA guidance on steps, obstacles and mistakes to avoid in the implementation of community mental health care | N | N | N | N | **N** | N |  |  |  | **N** | **N** |  |  |  |  |  |  |  |  |  |  |  |  |  |  |
| Ulloa, Rosa-Elena | 2018 | Effectiveness of a treatment guideline for schizophrenia in adolescents: Lessons from a middle-income country | N | N | N | N | **N** | N |  |  |  | **N** | **N** |  |  |  |  |  |  |  |  |  |  |  |  |  |  |
| Vancampfort, Davy | 2011 | Quality assessment of physical activity recommendations within clinical practice guidelines for the prevention and treatment of cardio-metabolic risk factors in people with schizophrenia | N | N | N | N | **N** | N |  |  |  | **N** | **N** |  |  |  |  |  |  |  |  |  |  |  |  |  |  |
| Velligan, Dawn I. | 2010 | Strategies for addressing adherence problems in patients with serious and persistent mental illness: Recommendations from the Expert Consensus Guidelines | Y | Y | Y | Y | **Y** | N | Y | Y | N | **N** | **N** |  |  |  |  |  |  |  |  |  |  |  |  |  |  |
| Verma, S. | 2017 | Ministry of Health clinical practice guidelines: schizophrenia | Y | Y | Y | Y | **Y** | Y | Y | Y | Y | **Y** | **Y** | Y | Y | N | Y | Y | **N** | Y | Y | N | Y | Y | **N** | **N** |  |
| von Malortie, S. | 2019 | [New national guidelines for the treatment of schizophrenia in Sweden] | Y | Y | Y | Y | **Y** | Y | Y | Y | Y | **Y** | **Y** |  |  |  |  |  | **N** |  |  |  |  |  | **N** | **N** | **Not in english** |
| Wallcraft, J. A. N. | 2011 | Partnerships for better mental health worldwide: WPA recommendations on best practices in working with service users and family carers | N | N | N | N | **N** | Y | N | N | Y | **N** | **N** |  |  |  |  |  |  |  |  |  |  |  |  |  |  |
| Waters, Flavie | 2017 | Delivering CBT for insomnia in psychosis: A clinical guide | N | N | N | N | **N** | N |  |  |  | **N** | **N** |  |  |  |  |  |  |  |  |  |  |  |  |  |  |
| Weinmann, Stefan | 2008 | Implementation of a schizophrenia practice guideline: Clinical results | N | N | N | N | **N** | N |  |  |  | **N** | **N** |  |  |  |  |  |  |  |  |  |  |  |  |  |  |
| **Note.**  **C**= Criteria **C1**= "Does the manuscript present a guideline?" **C2**= "Is the guideline specifically developed for the health condition of interest?; **C3**"Is the guideline a guideline on rehabilitation interventions?” **C4**= "Is the guideline Not older than 10 years?" **C5**= "Is it clear that there is No conflict of interest?" **C6**= "Is information on the strength of the recommendation provided?" | | | | | | | | | | | | | | | | | | | | | | | | | | | |
