# Supplementary material for: A systematic review of Clinical Practice Guidelines for the development of the WHO's Package of Interventions for Rehabilitation: focus on schizophrenia
Source: Front Public Health. 2023 Aug 15;11:1215617. doi: 10.3389/fpubh.2023.1215617 (PMC10465692; doi:10.3389/fpubh.2023.1215617)
Supplement: Supplementary file 2 [file Table_2.docx]

**Table 1. Classification of the strength of recommendation in the included clinical practice guidelines**

|  | **DGPPN** | **APA** | **NICE-a , NICE-b , NICE-c** | **SIGN** |
| --- | --- | --- | --- | --- |
| **Note** | - | **-** | - | The grade of recommendation relates to the strength of the evidence on which the recommendation is based. It does not reflect the clinical importance of the recommendation. |
| **Strong** | **A:** Strong recommendation (‘we recommend/we recommend not to’) 1++, 1+, (1-). | **Recommendation (denoted by number 1 after the guideline statement):** indicates confidence that the benefit of the intervention clearly outweigh harm. | **Strong:** For ‘strong’ recommendations on interventions that ‘should’ be used, the guideline development group is confident that, for the vast majority of people, the intervention (or interventions) will do more good than harm.  **Strong against:** For ‘strong’ recommendations on interventions that ‘should not’ be used, the guideline development group is confident that, for the vast majority of people, the intervention (or interventions) will do more harm than good. | **A:** At least one meta-analysis, systematic review, or RCT rated as 1++, and directly applicable to the target population; or a body of evidence consisting principally of studies rated as 1+, directly applicable to the target population, and demonstrating overall consistency of results.  **B:** A body of evidence including studies rated as 2++, directly applicable to the target population, and demonstrating overall consistency of results; or extrapolated evidence from studies rated as 1++ or 1+. |
|  |  |  |  |  |
| **Weak/Conditional** | **B:** Recommendation  (‘The GDG suggest/the GDG suggest not to’) 2++, 2+, (2-) or downgrading of 1++ / 1+ / (1-) due to methodological considerations.  **0:** Open recommendation (‘may/may not be considered’) 3, 4 or downgrading of 2++ / 2+ / (2-) due to methodological considerations. | **Suggestion (denoted by number 2 after the guideline statement):**  Indicates greater uncertainty. Although the benefits of statement are still viewed as outweighing the harms, the balance of benefit and harms are difficult to judge, or either the benefits or the harms may be less clear. | **Conditional**: For ‘conditional’ recommendations on interventions that should be ‘considered’, the guideline development group is confident that the intervention will do more good than harm for most patients. The choice of intervention is therefore more likely to vary depending on a person’s values and preferences, and so the healthcare professional should spend more time discussing the options with the patient. | **C:** A body of evidence including studies rated as 2+, directly applicable to the target population and demonstrating overall consistency of results; or extrapolated evidence from studies rated as 2++. **D:** Evidence level 3 or 4; or extrapolated evidence from studies rated as 2+. |
| **Expert opinion** | **GCP:** Clinical consensus (different strengths of recommendation possible). | - |  | **Good practice point (GPP):** recommended best practice based on the clinical experience of the guideline development group. |

**Table 2. Classification of the quality of the evidence in the included clinical practice guidelines**

|  | **DGPPN** | **APA** | **NICE-a , NICE-b , NICE-c** | **SIGN** |
| --- | --- | --- | --- | --- |
| **Note** | **-** | Many topics covered in this guideline have relied on forms of evidence such as consensus opinions of experienced clinicians or indirect findings from observational studies rather than research from randomized trials. | **-** | - |
| **High** | **1++:** High quality meta-analyses, systematic reviews of RCTs, or RCTs with a very low risk of bias.  **1+:** Well conducted meta-analyses, systematic reviews, or RCTs with a low risk of bias. | **A:** High confidence that the evidence reflects the true effect. Further research is very unlikely to change our confidence in the estimate of effect. | **High:** Further research is very unlikely to change our confidence in the estimate of effect. | **1++:** High quality meta-analyses, systematic reviews of RCTs, or RCTs with a very low risk of bias.  **1+:** Well conducted meta-analyses, systematic reviews, or RCTs with a low risk of bias. |
| **Moderate** | **1-:** Meta-analyses, systematic reviews, or RCTs with a high risk of bias.  **2++:** High quality systematic reviews of case control or cohort studies, High quality case control or cohort studies with a very low risk of confounding or bias and a high probability that the relationship is causal. | **B:** Moderate confidence that the evidence reflects the true effect. Further research may change our confidence in the estimate of effect and may change the estimate. | **Moderate:** Further research is likely to have an important impact on our confidence in the Estimate of effect and may change the estimate. | **1-:** Meta-analyses, systematic reviews, or RCTs with a high risk of bias.  **2++:** High quality systematic reviews of case control or cohort studies, High quality case control or cohort studies with a very low risk of confounding or bias and a high probability that the relationship is causal. |
| **Low** | **2+:** Well conducted case control or cohort studies with a low risk of confounding or bias and a moderate probability that the relationship is causal.  **2-:** Case control or cohort studies with a high risk of confounding or bias and a significant risk that the relationship is not causal. | **C:** Low confidence that the evidence reflects the true effect. Further research is likely to change our confidence in the estimate of effect and is likely to change the estimate. | **Low:** Further research is very likely to have an important impact on our confidence in the estimate of effect and is likely to change the estimate. | **2+:** Well conducted case control or cohort studies with a low risk of confounding or bias and a moderate probability that the relationship is causal.  **2-:** Case control or cohort studies with a high risk of confounding or bias and a significant risk that the relationship is not causal. |
| **Very low** | **3:** Non-analytic studies, e.g. case reports, case series.  **4**: Expert opinion. | - | **Very low:** Very uncertain about the estimate | **3:** Non-analytic studies, e.g., case reports, case series.  **4**: Expert opinion. |
